# Progress in Medicine

**Published:** 1895-06-01

**Authors:** 


### PROGRESS IN MEDICINE.

### NERVOUS DISEASES.

**Relation of Spinal Diseases to the Distribution and Lesions of the Blood Vessels of the Spinal Cord.**—R. T. Williamson[1] calls attention in an important contribution to the relation of a number of diseases of the spinal cord (1) to the distribution and (2) to the lesions of the spinal vessels. A transverse section of the cord may be mapped out into three districts according to the arterial supply: (1) the inner part of the grey matter supplied exclusively by the anterior median artery; (2) the superficial part of the white matter supplied by the peripheral arteries; (3) the remainder of the cord which receives arteries from both the peripheral and central systems; this area forms about one-third of the total transverse area of the cord. The blood supply may also be divided into (1) an anterior and (2) a posterior arterial system, and the transverse area of the cord may correspondingly be divided into two districts corresponding to the origin of supplying arteries from these two systems. In a case of poliomyelitis the affected area is practically that supplied by the anterior median arteries and branches. In a case of hereditary ataxia the affected area is that supplied by the peripheral arteries. In combined postero-lateral sclerosis the sclerotic area corresponds closely to that supplied by the posterior arterial system of the cord. In infantile paralysis the changes are found generally (1) around a branch of the commissural artery going to the anterior horn, or sometimes (2) around the anterior radicular arteries as they enter the anterior horns. The changes are found at any given level in one anterior horn only because the anterior median artery does not divide, but passes to one anterior horn only. In acute anterior poliomyelitis of the adult the pathology is probably the same as that of the disease in infants, but all the four extremities are much more frequently paralysed in the adult than in the infant. Sub-acute and chronic poliomyelitis are generally considered to have their commencement first in the ganglion cells of the anterior horn, but marked vascular changes are also found sometimes, if not always, to be present. Disseminated and transverse myelitis are also found to be associated in distribution with blood vessels which show marked distension of the perivascular sheath with round cells. In spinal syphilis the changes are mainly due to periphlebitis and periarteritis, while in locomotor ataxy there is in most cases no conclusive evidence as to the dependence of the cord lesions on vascular disease.

**Locomotor Ataxy.**—Nageotte[2] has made researches into the primary lesion of tabes under Professor Raymond's direction, and arrives at the conclusion that there is always in tabes a very intense perineuritis occupying that portion of the spinal nerves which is situated between the ganglion and the entrance of the roots into the arachnoid cavity; the process, at first embryonic, changing to fibroid at an advanced stage, is primary, and leads secondarily to degeneration of the posterior roots. These lesions involve also the anterior roots at the same place; but the nerve tubes of the latter resist the interstitial process much longer than the posterior roots, but in some cases must be regarded as the cause of the peripheral motor neuritis. Lagondaky[3] has minutely studied 212 cases in respect of a previous history of syphilis. Holding a middle position between Erb, who thinks syphilis an absolutely necessary antecedent, and Leyden, who thinks the syphilis must be left out of the ætiology of tabes, his investigations showed a proportion of 42 syphilitics per 100 cases of tabes. Letulle[4] showed at the Medical Society of the Hospitals in Paris a patient who had a perforating ulcer of the palate, accompanied by anasthesia of the whole mouth. This ataxic and syphilitic patient had suffered from perforating ulcer of the foot. Waldo[5] records a good example of Charcot's joint disease with perforating ulcer of the foot in a tabetic patient.

**Friedreich's Disease.**—H. J. Mackay[6] records four cases of this disease, three of the hereditary type occurring in one family, and one of the isolated type. Speaking of this last case, he points out the difficulty of certain diagnosis from chorea in the earlier stages of the disease. The difference between the two maladies is distinguished in (1) the distribution, (2) the nature of the twitchings, (3) the gait, (4) static equilibrium, (5) nystagmus, and (6) the state of the feet [the presence or absence of knee-jerk is highly important also.—*Abstractor*]. C. W. Burr[7] records a case with autopsy. The symptoms began to develop at the age of ten years, and the patient died of pulmonary tubercle. The cerebrum, cerebellum, pons and medulla were all small, the cord also smaller and of harder consistence than normal. The illustrations given of the sections of the cord show sclerosis of the posterior columns, direct and crossed pyramidal tracts, and direct cerebellar tract. The posterior nerve roots were also found to be degenerated, but the anterior were normal. As to the nature of the lesion, and as to whether the involvement of the lateral columns is a necessary factor, there is room for dispute, but the author thinks that a neurogliar sclerosis of the posterior columns is the primary lesion. Schultze[8] argues against Senator's hypothesis that Friedreich's ataxia depends upon an atrophy of the cerebellum, not on a systematic lesion of some of the nerve tracts in the medulla. Of the many cases described by Mendel, in only one was the affection of the medulla complicated by cerebellar atrophy.

**Amyotrophic Lateral Sclerosis.**—C. H. Brown[9] records a rare case in a boy aged fifteen. The symptons commenced three years before, there being gradually developed the phenomena of labio-glosso-laryngeal paralysis. This extended more or less rapidly to upper facial parts, to one sensory nerve (*i.e.*, the auditory), and proceeded down the body, implicating profoundly the muscles of respiration, and markedly those of the upper extremities. There is great atrophy in facial, lingual, throat, and respiratory muscles, and in those of the hand and arm. There is general arrest of development and great emaciation. Fibrillary twitchings, exaggeration of the tendon reflexes, and hyperspasticity of the tendons complete the *résumé* of the symptoms. Marinesco[10] at the Medical Society of the Hospitals in Paris reported his investigations into the pathological anatomy of a case of amyotrophy of the Charcot-Marie type, which begins in the feet and legs, and later involves the hands. In the spinal cord he found a systematised lesion of the posterior columns, resembling that of tabes; also a well-marked atrophic lesion of the anterior cornu corresponding to the amyotrophy, the peripheral nerves supplying the atrophied muscles were much degenerated, especially in the terminal portions. Senator[11] supports the view of Leyden (against that of Charcot), that amyotrophic lateral sclerosis cannot be separated from progressive bulbar paralysis, and the spastic phenomena are only a secondary feature of the disease. In a typical case, which died of "deglutition-pneumonia" marked atrophy was found in the ganglion cells of the anterior horns in the cervical and dorsal region; there were numerous hæmorrhages in both the grey and white matter, most abundant in the upper dorsal region, the lateral columns being not specially involved in this respect. There was no sclerosis of the lateral columns to explain the spastic symptoms. It is recommended that the term atrophic spastic paralysis be used as the name for the disease. Lannois and Lemoine[12] report a rare if not unique case. A woman developed weakness of the lower limbs with spasmodic movements; six months later total amaurosis supervened from atrophy of the optic nerves, not before reported to have occurred in amyotrophic lateral sclerosis; there were violent lightning pains, intense muscular contractions, and after three years and a-half medullary implications, shown by slight dysphagia and dyspnœa. Post-mortem there was found sclerosis of the lateral columns, of the medulla oblongata and of the optic nerves; the lesions of the pyramidal and cerebellar tracts were so intense as to have completely destroyed all the nerve fibres, wherein in ordinary cases some nerve fibres are always found intact. In the medulla the cells were comparatively unaffected, with the exception of the sensory nucleus of the pneumogastric and the nucleus of the hypoglossal nerves. The authors consider the case one of amyotrophic lateral sclerosis, in which the sclerosis was more intense than usual. The atrophy of the optic nerve they consider supports Stilling's theory that the nerve has a fourth root descending through the crus into the pons varolii.

**Anterior Poliomyelitis.**—Von Kahlden[13] reviews recent writings on the morbid anatomy with reference to its parenchymatous or interstitial origin. The author attaches great importance to the disappearance of the ganglion cells in groups, arguing that any lesion affecting these cells secondarily through their blood supply must be diffuse. He thinks there is no evidence why a non-corpuscular irritant circulating in the blood should involve only so small a part as the anterior horns of the spinal cord, many nerve fibrils being found intact in this condition. He maintains that the changes in the interstitial tissues do not need the assumption of an interstitial origin for the disease. Charcot and Dubil[14] record an example of the chronic type of this affection. Post-mortem were found, especially in the cervical region (1) endarteritis and sclerotic atrophy of the ganglion cells; (2) very slight alteration in the anterior roots, but well-marked degeneration of various peripheral nerves; and (3) in most muscles simple atrophy, but in those in which reaction of degeneration had been present there was wasting of the fibres.

**Paralysis Agitans.**—Redlich[15] records the lesions found in seven cases. Patches of sclerosis were found in the posterior columns of the cord, less extensive patches in the lateral columns, and few or none in the anterior. The changes were most marked in the cervical and lumbar bulbs. The sclerotic patches originated from the vessels and were of the nature of perivascular sclerosis. In the parts most affected the nerve-fibres had atrophied from the sclerosis. Williamson[16] gives a full *résumé* of the recent literature of paralysis agitans.

**Syringomyelia.**—C. L. Dana[17] records a case of syringomyelia, in which the symptoms resembled those of transverse myelitis. He thinks that acute or chronic transverse myelitis is frequently simulated by syringomyelia, and that the differentiation of disturbances in sensibility is not by any means always present in syringomyelia. His present case was due to glioma of the cord giving rise to hæmorrhages at intervals of time, and causing a considerable cavity within the cord. He strongly advocates the abandonment of the term syringomyelia, and the adoption of gliomatosis of the cord instead. Edgeworth[18] reports a very carefully-observed case of syringomyelia of the ordinary type, except that there was loss of tactile sensibility. Some of the symptoms of the case as described were known to have been in existence thirteen years previously.

[1] Med. Chron., Dec, 1894; Jan. and Feb., 1895. [2] Med. Week, Nov. 16, 1894. [3] Bull. Méd., quoted in Amer. Med.-Sur. Bull., Sept. 1, 1894. [4] Med. Week, July 27, 1894. [5] Brit. Med. Journ., Dec 1, 1894. [6] Amer. Journ. Med. Sciences, Aug. 1894. [7] University Med. Mag., quoted in Internat. Med. Mag., Aug., 1894. [8] Quoted in Edin. Med. Journ., Dec., 1894. [9] Journ. Ment. and Nerv. Dis., Nov., 1894. [10] Med. Week, July 27, 1894. [11] Deutsch. Med. Woch., May 17, 1894. [12] Med. Week, July 27, 1884. [13] Centralb. f. allg. Path., quoted in Epitome B. M. J., Oct. 20, 1894. [14] Progrès Médical, quoted in Lancet, July 28, 1894. [15] Neurol. Centralbl., July, 1894, [16] Med. Chron., Aug., 1894. [17] Journ. Nerv. and Ment. Dis., Oct., 1894. [18] Bristol Med.-Chir. Journ., June, 1894.

